# Temperature Observation Time and Type Influence Estimates of Heat-Related Mortality in Seven U.S. Cities

**DOI:** 10.1289/ehp.1509946

**Published:** 2015-12-04

**Authors:** Robert E. Davis, David M. Hondula, Anjali P. Patel

**Affiliations:** 1 Department of Environmental Sciences, University of Virginia, Charlottesville, Virginia, USA; 2 Center for Policy Informatics, and School of Geographical Sciences and Urban Planning, Arizona State University, Phoenix, Arizona, USA

## Abstract

**Background:**

Extreme heat is a leading weather-related cause of mortality in the United States, but little guidance is available regarding how temperature variable selection impacts heat–mortality relationships.

**Objectives:**

We examined how the strength of the relationship between daily heat-related mortality and temperature varies as a function of temperature observation time, lag, and calculation method.

**Methods:**

Long time series of daily mortality counts and hourly temperature for seven U.S. cities with different climates were examined using a generalized additive model. The temperature effect was modeled separately for each hour of the day (with up to 3-day lags) along with different methods of calculating daily maximum, minimum, and mean temperature. We estimated the temperature effect on mortality for each variable by comparing the 99th versus 85th temperature percentiles, as determined from the annual time series.

**Results:**

In three northern cities (Boston, MA; Philadelphia, PA; and Seattle, WA) that appeared to have the greatest sensitivity to heat, hourly estimates were consistent with a diurnal pattern in the heat-mortality response, with strongest associations for afternoon or maximum temperature at lag 0 (day of death) or afternoon and evening of lag 1 (day before death). In warmer, southern cities, stronger associations were found with morning temperatures, but overall the relationships were weaker. The strongest temperature–mortality relationships were associated with maximum temperature, although mean temperature results were comparable.

**Conclusions:**

There were systematic and substantial differences in the association between temperature and mortality based on the time and type of temperature observation. Because the strongest hourly temperature–mortality relationships were not always found at times typically associated with daily maximum temperatures, temperature variables should be selected independently for each study location. In general, heat-mortality was more closely coupled to afternoon and maximum temperatures in most cities we examined, particularly those typically prone to heat-related mortality.

**Citation:**

Davis RE, Hondula DM, Patel AP. 2016. Temperature observation time and type influence estimates of heat-related mortality in seven U.S. cities. Environ Health Perspect 124:795–804; http://dx.doi.org/10.1289/ehp.1509946

## Introduction

Heat is a primary weather-related cause of human mortality in the United States (Centers for Disease Control and Prevention 2006). Numerous studies have demonstrated that mortality increases significantly when some location-specific, temperature-based threshold is exceeded (Gosling et al. 2009; Hajat and Kosatsky 2010). The statistical robustness of this relationship has prompted the implementation of heat-warning systems and other intervention strategies in many localities to mitigate heat mortality that is largely preventable (Ebi and Schmier 2005; Smoyer-Tomic and Rainham 2001).

Despite the growth of this research area as well as the interest arising from a potential increase in heat-related mortality from higher thermal exposure in the future, few researchers have systematically examined how the time of weather observation influences the heat–mortality relationship. Prior studies of heat-related mortality have considered lag effects to account for the time between the onset of debilitating conditions and the resulting mortality event (Braga et al. 2001; Davis et al. 2003a). A few studies have compared different exposure variables to determine whether there is an optimal variable to incorporate in heat-mortality models (Barnett et al. 2010; Zhang et al. 2012). However, with few exceptions (e.g., Hondula et al. 2012), no one has explicitly examined if the observation time of the independent variable (i.e., the heat metric being evaluated as the risk factor of interest), or the manner in which it is calculated, significantly influences the weather–mortality relationship.

Daily maximum temperature is a common metric in heat-mortality studies because it is a proxy for the maximum thermal stress on the body (Hajat et al. 2006; Tan et al. 2007). Although maximum temperature typically occurs a few hours after peak sunlight intensity, on days with frontal passages, it can occur at any time of day (Bristow and Campbell 1984; Karl et al. 1986). In contrast, the physiological rationale for using minimum temperature is that, during heat wave periods with high minima, the body does not have the opportunity to sufficiently recover from the thermal stresses experienced during the previous day (Le Tertre et al. 2006; Schwartz 2005). Minimum temperature typically occurs just after sunrise, but based upon air mass changes and cloud cover, the hour of minimum temperature also varies (Bristow and Campbell 1984; Karl et al. 1986). The pattern of daily warming and cooling (between maximum and minimum temperatures) is neither symmetric nor consistent, and by using maximum and minimum temperatures in epidemiological models, the researcher implicitly accepts that the exposure (independent) variable is being sampled at different times of the day.

Environmental health researchers apply different conceptualizations of maximum and minimum temperature in temperature–mortality associations, with some (e.g., Medina-Ramón and Schwartz 2007) selecting maximum and minimum temperature based on the highest and lowest values of 24 hourly observations taken at the “top” of each hour, whereas others (e.g., Guo et al. 2013) use readings from maximum–minimum thermometers, in which the maximum and minimum may occur at any time (i.e., the extreme readings need not align with the hourly observations). To avoid possible statistical biases that can occur with shifts in the sampling time of maximum and minimum temperature, some researchers have selected specific times of day to characterize exposure [e.g., 0700 or 1400 hours local standard time (LST)] using the times typically associated with the daily high or low (e.g., Davis et al. 2003b). Another commonly used thermal variable is daily mean temperature (e.g., Donaldson et al. 2003; Huynen et al. 2001), but even this variable can be computed in a variety of ways, including the average of the maximum and minimum (which are temporally variant) and the average of hourly readings. Thus, the choice of observation time is seemingly given little consideration in the overall study design but it has an inherent influence on the exposure variable used in the research.

A variety of indices and human comfort models have been developed that incorporate factors in addition to air temperature that influence the human heat load, including radiative and convective heat exchange and heat loss via evaporation (Gosling et al. 2009; Jendritzky and Tinz 2009; Koppe et al. 2004), but these models require more input variables. In the United States, the most commonly used index is apparent temperature (AT, also known as the Heat Index), a variable that combines temperature with humidity (and a minor wind correction) (Steadman 1984). Several systematic comparisons have been conducted between heat mortality associations and different exposure metrics. City- and national-scale studies have most frequently reported that daily maximum or mean summary variables (either air temperature or AT) produce stronger relationships than daily minima (Anderson and Bell 2009; Armstrong et al. 2011; Baccini et al. 2008). There is less agreement regarding the performance of air temperature versus AT and other more complex daily summary metrics such as air mass types (Hajat et al. 2010; Metzger et al. 2010; Zhang et al. 2014), and there is some evidence that optimal variable selection varies by location, season, and age cohort (Barnett et al. 2010; Conti et al. 2005). None of these studies, however, have considered observation time and type of temperature observation, which could also materially influence temperature–mortality associations.

The purpose of this research is to determine whether variations in air temperature observation time and type impact the resulting relationship to heat-related mortality. For example, would different mortality relationships result if 0700 hours temperature is used as the exposure variable versus 1500 hours temperature or a daily mean temperature? Does the method used to calculate daily mean temperature materially influence the results? Is either maximum or minimum temperature more closely associated with heat mortality? To our knowledge, no one has explicitly examined the relationship between hour-by-hour variations in temperature and heat-related mortality. If these factors do vary significantly, then this information could inform variable selection when attempting to estimate the burden of extreme heat on human health and help guide the development and deployment of certain adaptation strategies for coping with health impacts.

## Methods

Daily mortality frequencies were acquired from the departments of health in seven cities: Atlanta, Georgia; Boston, Massachusetts; Minneapolis-St. Paul, Minnesota; Philadelphia, Pennsylvania; Phoenix, Arizona; Seattle, Washington; and St. Louis, Missouri. These cities were selected because they represent a range of climates (Table 1) and heat-mortality responses and their heat-mortality relationships have been studied extensively as part of a larger project (Hondula et al. 2014, 2015; Saha et al. 2014). Boston, Minneapolis, Philadelphia, Atlanta, and St. Louis are all located in the eastern half of the United States where moist tropical days regularly occur during the summer months; dry tropical days are less common but also occur. The frequency and intensity of these synoptic-scale occurrences vary, with Atlanta and St. Louis being most commonly exposed and Boston and Minneapolis experiencing more moderate but still potentially dangerous heat events. Seattle is located in the Pacific northwestern region and generally experiences mild and dry summer weather, with occasional heat waves that occur when high pressure stagnates over the region. These events represent a significant deviation from normal weather experienced in the region. Phoenix is located in the semiarid southwest region of the country, and its climate is characterized by an intense dry heat that lasts for nearly the entire warm season. Phoenix occasionally deals with periods of elevated humidity conditions in the late summer months when regional wind patterns shift to transport moisture northward from the Gulf of Mexico, Gulf of California, and/or eastern tropical Pacific Ocean.

**Table 1:** Locations and data sources.

City (abbreviation) and geographic centroid	Climate classification (Köppen type)^*a*^	Summer^*b*^ maximum^*c*^ temperature range (median)	Geographic area	Population of study area in 2000	Mortality data source	Period of record	Average number of deaths per year	Weather data missing	Weather station location
Atlanta (ATL) 33.875°N 84.301°W	Consistently hot, humid summers (Cfa)	16.7–40.0°C (31.1°C)	4,807 km^2^	2,810,278	Georgia Dept. of Community Health	1994–2007 (14 years)	15,242	3.4%	Hartsfield Jackson Atlanta International Airport 33.637°N, 84.428°W
Boston (BOS) 42.392°N 71.102°W	Mild but humid summers with periodic hot spells (Dfa)	11.7–38.3°C^*d*^ (26.7°C)	724 km^2^	1,536,926	Massachusetts Dept. of Public Health	1987–2007 (21)	13,047	1.0%	General Edward Lawrence Logan International Airport 42.363°N, 71.006°W
Minneapolis (MSP) 44.956°N 93.197°W	Mild summers with periodic hot spells (Dfa)	11.7–38.3°C^*d*^ (27.3°C)	3,637 km^2^	2,265,814	Minnesota Center for Health Statistics	1992–2008 (17)	13,715	1.0%	Minneapolis-St. Paul International Airport 44.882°N, 93.222°W
Philadelphia (PHL) 40.011°N 75.134°W	Warm but variable summers with annual heat waves (Cfa)	15.0–39.4°C (30.0°C)	352 km^2^	1,509,525	Pennsylvania State Dept. of Health	1983–2008 (26)	15,752	3.1%	Philadelphia International Airport 39.872°N, 75.241°W
Phoenix (PHX) 33.563°N 112.030°W	Hot and arid with occasional periods of high summer humidity (BSh)	23.9–50.0°C (41.1°C)	5,357 km^2^	2,944,227	Arizona Dept. of Health Services	1989–2007 (19)	19,117	0.3%	Phoenix Sky Harbor International Airport 33.434°N, 112.012°W
St. Louis (STL) 38.614°N 90.459°W	Warm to hot and humid summers (Cfa)	14.4–41.7°C (31.1°C)	1,751 km^2^	1,401,298	Missouri Dept. of Health	1980–2008 (29)	13,681	2.4%	Lambert St. Louis International Airport 38.747°N, 90.361°W
Seattle (SEA) 47.536°N 122.259°W	Mild, dry summers with minimal rainfall (Csb)	11.7–37.8°C (22.8°C)	1,071 km^2^	1,567,483	Washington State Dept. of Health	1988–2009 (22)	10,833	0.8%	Seattle-Tacoma International Airport 47.449°N, 122.309°W
^***a***^Climate types are extracted from a 0.5° resolution map of the updated Köppen–Geiger classification developed by Kottek et al. (2006). ^***b***^Based on June–August observations during the study period. ^***c***^Where maximum is considered the highest temperature recorded during the day regardless of time (the metric defined as max in Table 2). ^***d***^The observed summer maximum temperature ranges for these two cities during the study period are, in fact, identical.									

The periods of record vary between cities based on the availability of health records, with Atlanta having the fewest years of data (14 years) and St. Louis having the most (29 years) (Table 1). Mortality data were provided at the ZIP Code Tabulation Area (ZCTA) scale, and these daily counts were aggregated for the 48–101 ZCTAs that encompassed each metropolitan area (U.S. Census Bureau 2012).

**Figure 1:**
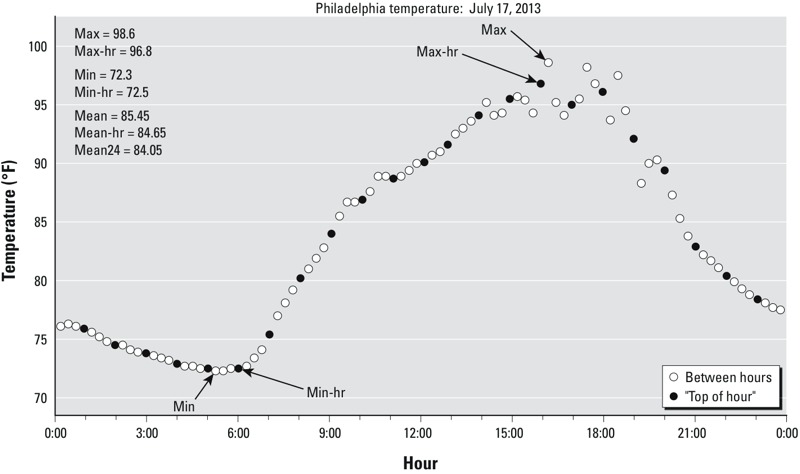
Example demonstrating the calculation of different temperature variables during a high heat event in Philadelphia, Pennsylvania (17 July 2013). Each observation is shown (open circle) along with the hourly observation taken closest to the “top” of each hour (closed circle). Variable definitions: max, min: highest and lowest temperature between midnight and midnight regardless of observation time; max-hr, min-hr: highest and lowest temperature of the 24 observations made over the course of a day that occur near the top of each hour; mean: the average of max and min; mean-hr; the average of max-hr and min-hr; mean24: the average of 24 hourly observations made at the top of each hour.

Hourly temperature data from all calendar months were acquired from a first-order weather station in each metropolitan area (Table 1). Missing observations, which accounted for no more than 3.4% of all observations (Table 1), were linearly interpolated from the nearest values using the linear interpolation function in SPSS (version 21; SPSS, IBM). The large majority of interpolated observations were isolated missing values. Here, “hourly” refers to observations taken at least once per hour, but often at a much higher resolution. The measurement frequency varies over time as National Weather Service measurement protocols have changed. As an example, a 24-hr period during a high heat event in Philadelphia is shown in Figure 1 in which observations were taken four times per hour. Maximum (max) and minimum (min) temperature were determined from the extremes observed each day (Table 2)—these observations are free to occur at any time of the day (in Figure 1, e.g., max and min occur at 1615 and 0515 hours, respectively). These were compared with the maximum and minimum temperatures acquired from the observation taken nearest the “top” of each hour (max-hr and min-hr) (Figure 1 and Table 2). Daily mean temperature was calculated in three different ways: as the average of max and min (mean), the average of max-hr and min-hr (mean-hr), and the average of 24 measurements taken at the top of each hour (mean24) (Table 2). So in this example, because of the long period of consistent low temperatures before sunrise, the actual minimum temperature (min) was close to the hourly minimum temperature (min-hr), but the actual maximum was almost 2°F higher than the hourly value (Figure 1). These differences influenced the calculation of daily mean temperature, which on this day varied by 1.4°F, depending upon the formulation used.

**Table 2:** Temperature variables used in heat-mortality modeling.

Variable abbreviation	Description
Max	Maximum temperature between midnight and midnight as recorded by a maximum/minimum thermometer. Can occur at any time.
Max-hr	Maximum temperature of 24 hourly values taken at the observations nearest the top of each hour (e.g., midnight, 0100 hours).
Min	Minimum temperature between midnight and midnight as recorded by a maximum/minimum thermometer. Can occur at any time.
Min-hr	Minimum temperature of 24 hourly values taken at the observations nearest the top of each hour (e.g., midnight, 0100 hours).
Mean	(Max + min)/2.
Mean-hr	[(Max-hr) + (min-hr)]/2.
Mean24	Average of 24 hourly values taken at the top of each hour.

Each city’s mortality time series was modeled using a generalized additive model of the form:

Log(M) = *s*(time, *k* = *y* × *a*) + *s*(temp, *k* = 4), [1]

where M is the daily mortality count, *s* represents a fixed thin-plate regression spline with *k* – 1 degrees of freedom, “time” is an integer counter for each day in the period of record, *y* represents the number of years in each city’s period of record, *a* is the number of degrees of freedom used for modeling the “time” term for each year (we used 7 degrees of freedom per year for the main analysis and 5 degrees of freedom per year as a sensitivity analysis), and “temp” represents the daily temperature time series as measured either at each hour or for one of the formulations of maximum, minimum, or mean temperature shown in Table 2 (following, e.g., Anderson and Bell 2009). The model used a quasi-Poisson link function to account for overdispersion in the mortality data and was constructed with year-round temperature and mortality data.

We ran separate models to estimate relative risks (RRs) for each exposure variable on the day of death (lag 0) and each of the three lag days (lag 1–3), including the seven daily temperature exposure metrics (Table 2) and 24 hourly temperature measurements on each day. Mortality is archived at daily intervals, so a mortality event recorded on a day with extreme heat is characterized as lag 0, mortality on the day following high heat is lag 1, and so on. In one analysis, the seven daily summary temperature metrics are compared with the RR of mortality for each day. Additionally, an hourly analysis is performed in which the RR of mortality is modeled separately for each hour. Only the independent variable (air temperature) varies for a given day—mortality varies daily but not hourly. Temperature percentiles were defined separately for each different independent variable and based on data from the entire calendar year. The 85th percentile corresponds to approximately the 55th hottest day of an average year, or roughly the median summer temperature for a 3-month warm season.

Each RR represents the estimated risk of mortality at the 99th percentile of the metric-specific temperature distribution (M_99_) relative to the 85th percentile (M_85_):

RR = e^M_99_ – M_85_^. [2]

**Figure 2:**
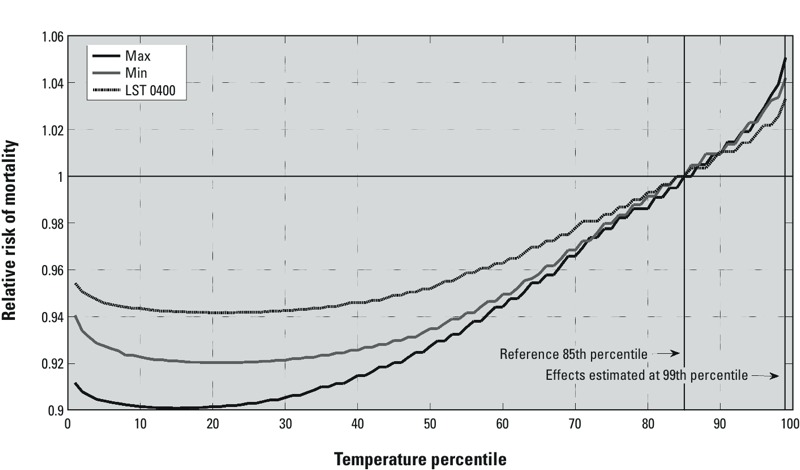
Modeled relative risks comparing mortality at each percentile of the temperature range for three different temperature metrics to mortality at the 85th percentile of the temperature range. The relative risks are shown for three different same-day (lag 0) temperature metrics for Philadelphia. The solid black line shows the relative risks for daily maximum temperature (max); solid gray for daily minimum temperature (min); dark dashes for 0400 hours local standard time (LST) temperature. Percentiles are calculated separately for each temperature metric. Vertical lines indicate temperature percentiles used in the calculation of relative risks and confidence intervals. Here, the relative risk of mortality for the 99th versus 85th percentile of each temperature metric is estimated at 1.051 for max, 1.042 for min, and 1.033 for temperature at 0400 hours LST.

For example, in Philadelphia, the estimated RR of mortality for the 99th percentile of maximum temperature on lag 0 (max) was 1.051, whereas the estimated RR for the 99th percentile of minimum temperature on lag 0 (min) and of hourly temperature measured at 0400 hours LST were 1.042 and 1.033, respectively (Figure 2).

The confidence interval (CI) for each RR was calculated using the equation


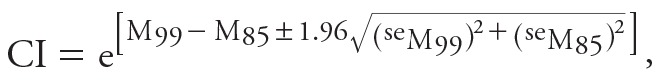
,[3]

where “se” is the standard error for mortality predictions at a given temperature percentile (Gardner and Altman 1989). Statistical significance was determined when the CI for a given temperature effect did not include one. (As a sensitivity analysis, we also estimated RRs comparing the estimate risk at the 99th percentile to the 90th percentile, instead of using the estimated risk at the 85th percentile as the reference value.) We also extracted a generalized cross-validation score for each model to use as a diagnostic of model fit (Wood 2006).

Analyses were run using the *mgcv* package (version 1.7-6) with R version 2.13.2 (R Core Team 2012; Wood 2006). Model-based estimates of the risk of mortality and corresponding standard errors for the 99th and 85th percentiles of each temperature metric’s distribution were extracted using the “terms” feature of the *predict* function.

## Results

**Figure 3:**
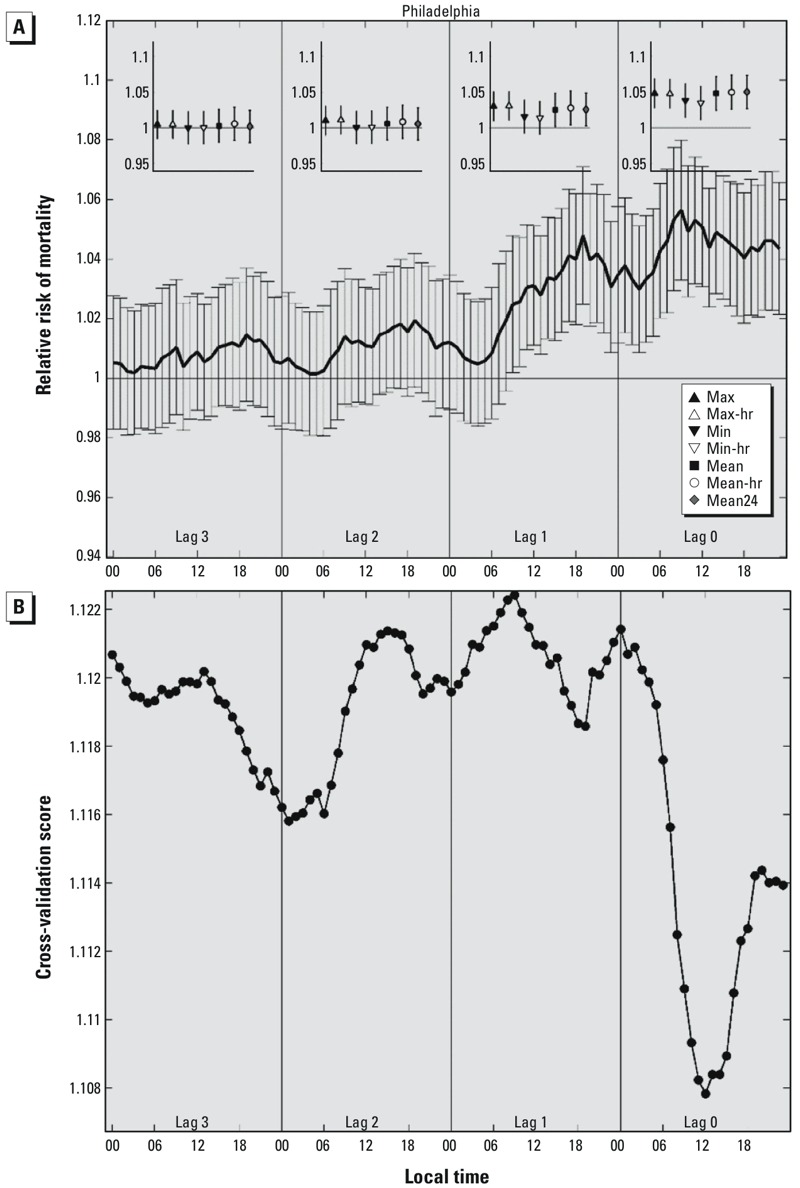
(*A*) The main panel shows the relative risk of mortality (with 95% confidence intervals) in Philadelphia for temperatures at the 99th percentile compared with the 85th percentile estimated using separate models for temperatures at each hour and on each lag day, where lag 0 represents the day of death, lags 1–3 represent the 1, 2, and 3 days before the day of death. Inset panels show RRs for the seven daily temperature metrics on each lag day: max = maximum temperature; max-hr =maximum hourly temperature; min = minimum temperature; min-hr = minimum hourly temperature; mean = (max + min)/2; mean-hr = (max-hr + min-hr)/2; mean24 = average value of the 24 hourly temperatures. All models are adjusted for time trends (7 df). (*B*) Cross-validation scores for each of the hourly models examined in *A*; lower cross-validation scores indicate better model fit.

**Figure 4:**
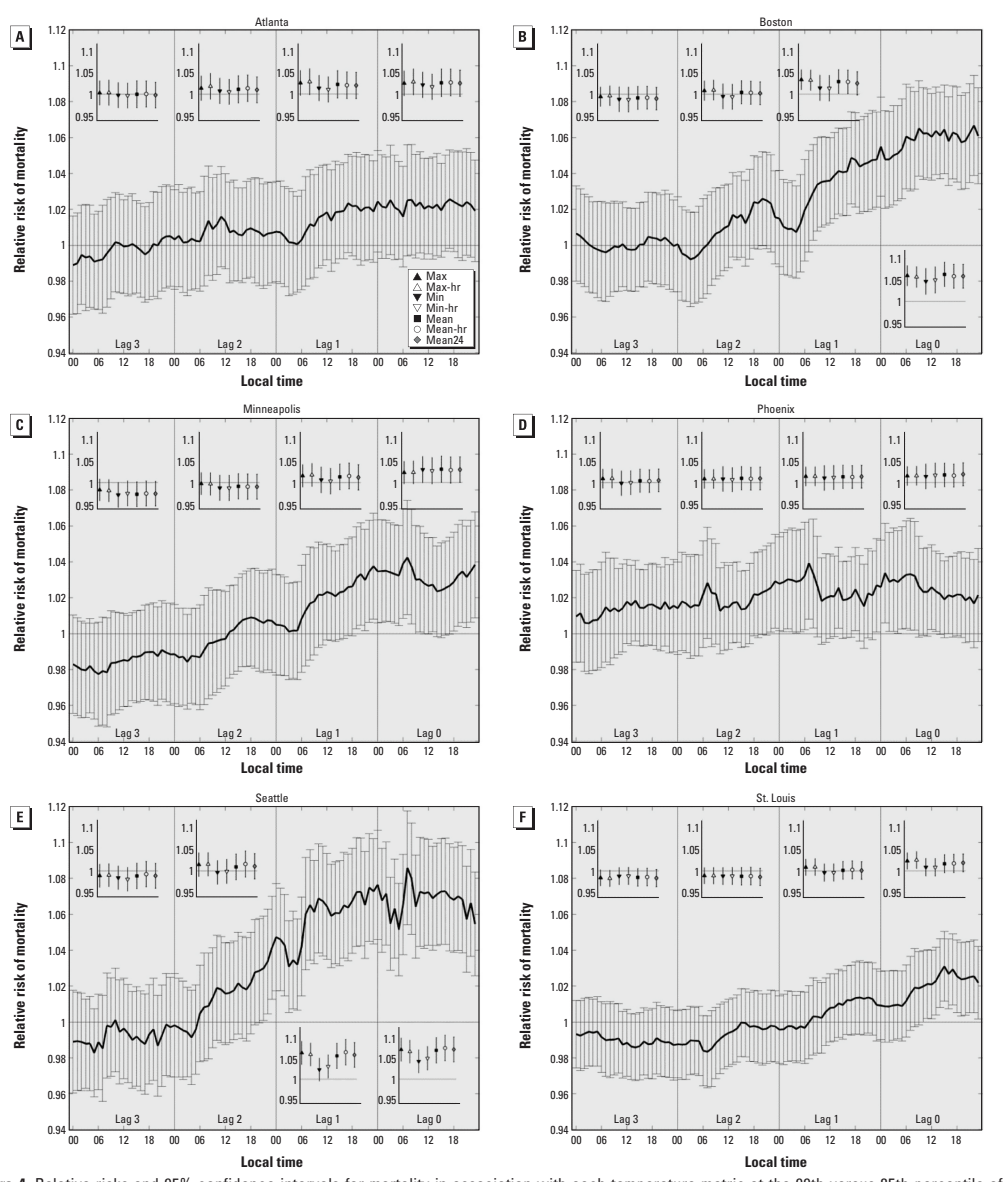
Relative risks and 95% confidence intervals for mortality in association with each temperature metric at the 99th versus 85th percentile of the temperature distribution for (*A*) Atlanta, (*B*) Boston, (*C*) Minneapolis, (*D*) Phoenix, (*E*) Seattle, and (*F*) St. Louis, respectively. Each relative risk is estimated using a separate model for temperatures at each hour and on each lag day, where lag 0 represents the day of death, lags 1–3 represent the 1, 2, and 3 days before the day of death. Inset panels show relative risks for the seven daily temperature metrics on each lag day: max = maximum temperature; max-hr =maximum hourly temperature; min = minimum temperature; min-hr = minimum hourly temperature; mean = (max + min)/2; mean-hr = (max-hr + min-hr)/2; mean24 = average value of the 24 hourly temperatures. The key for the daily metrics is shown in *A*. All models are adjusted for time trends (7 df).

Statistically significant relationships between temperature and mortality were estimated for each city except Atlanta. The strength of these relationships varied markedly, and often systematically, when comparing across cities, temperature metrics, and lag times (Figures 3 and 4).

As an example, estimated RRs for Philadelphia are presented in Figure 3. RRs for the estimated risk of mortality at the 99th versus 85th percentile of the hourly temperature distribution were statistically significant beginning at 0900 hours LST on the day before death (lag 1, 0900 hours LST RR = 1.025; 95% CI: 1.002, 1.048) through every hour of the day of death (lag 0, 2300 hours LST RR = 1.044; 95% CI: 1.021, 1.066). However, the strongest temperature–mortality relationships were evident at 0900 hours LST on lag 0 (RR = 1.056; 95% CI: 1.033, 1.080) and at 1900 hours LST on lag 1 (RR = 1.048; 95% CI: 1.025, 1.071). On each lag day, RRs were lowest during the early morning hours (after midnight through approximately 0600 hours LST) and highest during the late morning through late evening hours (approximately 0900–2200 hours LST), suggesting a diurnal pattern of heat impacts. However, patterns of RRs for hourly temperatures on the day of death (lag 0) should be interpreted with caution because temperature “exposures” measured later in the day are increasingly likely to have occurred after the time of death, and positive associations are therefore more likely to reflect correlations of hourly temperatures later in the day with temperatures earlier in the day, rather than actual effects of hourly heat on mortality. We include the full 24 hr on lag 0 for completeness, but these late-day relationships should be considered with caution.

RRs for mortality at the 99th versus 85th percentile of each of the seven daily temperature metrics were broadly consistent with the hourly RRs. For example, in Philadelphia, RRs were statistically significant for all daily exposure metrics on lag 0 (Figure 3A, inset). However, by lag 1, RRs for the two minimum temperature metrics (min and min-hr) were no longer significant, and none of the daily metrics were significant predictors of mortality by lag 2. Cross-validation scores computed for the hourly variables for Philadelphia (Figure 3B) show that model fit was strongest when temperatures near midday were used from lag 0, close to the observation time associated with the largest effect size. Model performance deteriorated rapidly between lag 0 and lag 1.

In Boston and Seattle, there were statistically significant relationships throughout the day of lag 0 and most (or all) of the hours of lag 1 (Figure 4B,E). In both cities, the relationship was markedly weaker in the early morning hours of lag 1, but in the case of Seattle, these relationships were still significant. Thus, the general pattern of diurnality observed in Philadelphia was also present in Boston and Seattle: The mortality–temperature relationships tended to be weaker in the late overnight hours and strongest between late morning and evening. These hourly results were consistent with the daily summary variables—whereas RRs for at least two of the three mean temperature metrics (mean, mean-hr, and mean24) and both maximum temperature metrics (max and max-hr) were still significant on lag 1 for both cities, one or both of the minimum temperature metrics was not. It is noteworthy that at lag 3 for Seattle, all RRs were negative for the daily metrics and most of the hourly metrics, a pattern that might reflect “mortality displacement,” the idea that reduced mortality rates may occur 72–96 hr after a major heat event that hastens the time of death in frail individuals (Hajat et al. 2005; Saha et al. 2014). However, evidence of mortality displacement must be interpreted with caution in the absence of information on the length or severity of each heat event.

A different pattern of mortality–temperature relationship was evident in Minneapolis and St. Louis (Figure 4C,F). In these cities, a few hours at lag 0 had weak (but statistically significant) relationships, but almost none were evident by lag 1 and later. In Minneapolis, the strongest association with hourly temperatures was at 0700 hours LST on lag 0 (RR = 1.042, 95% CI: 1.011, 1.074), and associations with daily mean and daily minimum temperature metrics were slightly stronger than RRs for the maximum temperature metrics on lag 0. In St. Louis, hourly RRs were most positive in the afternoon of lag 0 as compared to the morning, when the RRs decline. The overall pattern on lags 0 and 1 suggests a diurnal relationship similar to that found in Boston and Seattle, even though hourly RRs were significant after noon on lag 0 only, and all hourly RRs were < 1 on lags 2 and 3. The relationship was strongest in St. Louis in the afternoon on lag 0 (RR = 1.031; 95% CI: 1.012, 1.050 at 1500 hours LST) and for both maximum temperature variables compared with mean or minimum temperatures. Negative RRs for hourly temperatures and mortality on lags 2 and 3 in Minneapolis and St. Louis might reflect mortality displacement, though RRs are close to the null, and associations between temperature and mortality on lag 0 were generally weak for St. Louis.

We were unable to detect a significant mortality–temperature relationship for Atlanta using all-cause mortality records (Figure 4A). Neither the hourly nor summary variables were clearly related to mortality in Atlanta.

In Phoenix, although all hourly RRs on all lag days were positive, only a few were statistically significant (Figure 4D). Between lag 1 and lag 0 as well as lag 2 and lag 1, RRs were larger for the overnight and morning hours from roughly 2000 hours LST to 0800 hours LST, suggesting a relationship that differs from the other six cities. None of the daily summary variables were significantly associated with mortality in Phoenix. So unlike Philadelphia, Boston, and Seattle, where RRs showed evidence of a diurnal pattern, the strongest mortality–temperature models for Phoenix are constructed using morning temperatures for specific hours.

In general, for the six cities with significant relationships, RRs for daily minimum temperature (min and min-hr) were closer to the null than RRs for the daily maximum and mean temperature metrics, though differences were generally small and limited to lag 0 and lag 1. Some subtle differences were evident based on calculation/observation method for the daily summary variables. In Boston, for example, the RR for lag 1 mean temperature was estimated as 1.030 (95% CI: 1.002, 1.058), for lag 1 mean-hr as 1.029 (95% CI: 1.002, 1.056), and for lag 1 mean24 as 1.025 (95% CI: 0.998, 1.052)—so mean and mean-hr were significant but mean24 was not. Differences in RR estimates were similarly small for the maximum and minimum metrics. The confidence intervals, which indicate the precision of effect estimates, exhibited similar widths for the different methods of calculating mean, maximum, and minimum temperatures.

**Figure 5:**
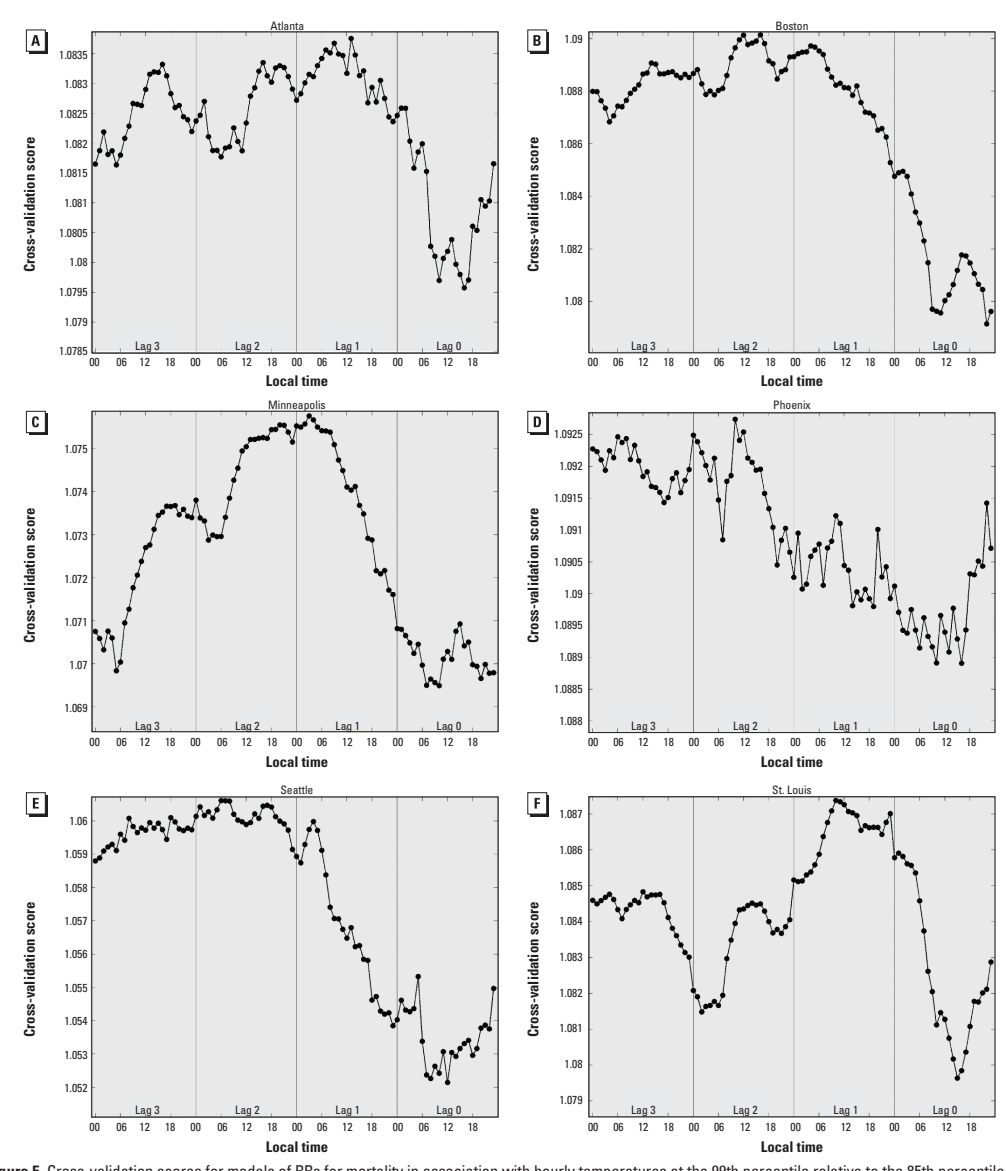
Cross-validation scores for models of RRs for mortality in association with hourly temperatures at the 99th percentile relative to the 85th percentile, estimated using separate models for each hour and lag day, adjusted for time trends using a time spline with 7 degrees of freedom per year. (*A*) Atlanta, (*B*) Boston, (*C*) Minneapolis, (*D*) Phoenix, (*E*) Seattle, and (*F*) St. Louis. Lower cross-validation scores indicate better model fit.

Cross-validation scores for models using hourly temperature observations were consistently lowest on lag 0 compared with lags 1–3 (Figure 5). The times of best model fit commonly occurred during the daytime hours as opposed to overnight, with most cities showing better model fit in the late morning than in the afternoon. Minimum cross-validation scores did not uniformly align with the times associated with the largest RR.

## Discussion

Evidence for stronger temperature–mortality relationships in northern cities and weaker or nonsignificant models in southern cities is consistent with previous research indicating that cooler locations are more susceptible to heat impacts (Kalkstein and Davis 1989; Medina-Ramón and Schwartz 2007), possibly because of reduced acclimatization or adaptation (Davis et al. 2003a; Gosling et al. 2009). Identification of the strongest associations between temperature and mortality on the day of the heat event or on the following day is likewise consistent with prior findings (Davis et al. 2003b; Gosling et al. 2009). However, to the best of our knowledge, no previous research has documented the hourly variability in the strength of temperature–mortality relationships, nor has there been any comparison of daily summary temperature variables computed using different methods. Here, we show that the selection of time-of-day and summary measure for the independent variable can influence the strength of the temperature–mortality association in the resulting model.

In three of the seven study cities (Boston, Philadelphia, and Seattle), associations between mortality and hourly temperatures during the daytime and evening were stronger than associations with hourly temperatures during the morning hours, and associations between mortality and daily mean or maximum temperature metrics were slightly stronger than RRs based on daily minimum temperature metrics. Thus, warm days and evenings had a greater mortality impact in these cities than warm nights. Contrasting theories on heat mortality emphasize either the impact of extreme conditions that focus on the physiological stress placed on the body or the lack of a period of early morning respite during heat waves when the body recovers from daily thermal stress (Basu and Samet 2002; Braga et al. 2002; Koppe et al. 2004). In Philadelphia, for example, mortality relative risk estimates for 2000 hours LST on lag 1 were 4% higher for the 99th versus the 85th percentile of temperature (RR = 1.040; 95% CI: 1.017, 1.062). However, there is no relative increase in mortality at 0400 hours LST on lag 1 (RR = 1.005; 95% CI: 0.984, 1.026). This morning–daytime contrast in the estimated temperature effect could occur because morning temperatures are not strong indicators of the days with the highest peak heat stress, a proposition supported by the correlation coefficients shown in Table 3. For example, the correlation between minimum temperatures (which tend to occur in the morning) and maximum temperatures (which tend to occur in the afternoon) was no higher than 0.577 in any city for days with mean temperatures above the 85th percentile of the annual mean temperature distribution. Additionally, morning temperatures may be less consequential for mortality impacts than afternoon temperatures because the relatively cooler morning conditions provide some protective effect and/or people’s behaviors and activities in the mornings are less likely to lead to heat stress.

**Table 3:** Correlation coefficients between selected pairs of temperature observations for each city (abbreviations in Table 1).

Temperature metrics compared/time span of comparison	Atlanta	Boston	Minneapolis	Philadelphia	Phoenix	Seattle	St. Louis	All-city average
1200 & 1800 hours LST
Entire summer^*b*^	0.713	0.863	0.859	0.829	0.838	0.882	0.818	0.829
Days ≥ 85th percentile^*c*^	0.498	0.629	0.644	0.594	0.598	0.772	0.568	0.615
1200 hours LST & mean^*a*^
Entire summer	0.883	0.933	0.901	0.911	0.888	0.934	0.912	0.909
Days ≥ 85th percentile	0.793	0.828	0.795	0.805	0.690	0.884	0.812	0.801
Max^*a*^ & min^*a*^
Entire summer	0.655	0.758	0.733	0.705	0.645	0.634	0.783	0.702
Days ≥ 85th percentile	0.423	0.472	0.535	0.376	0.143	0.395	0.577	0.417
Mean & mean24^*a*^
Entire summer	0.992	0.997	0.995	0.996	0.993	0.995	0.997	0.995
Days ≥ 85th percentile	0.974	0.991	0.988	0.986	0.975	0.989	0.990	0.985
Max lag 0 & max lag 1
Entire summer	0.755	0.558	0.677	0.659	0.724	0.692	0.672	0.677
Days ≥ 85th percentile	0.660	0.341	0.459	0.484	0.553	0.457	0.456	0.487
^***a***^Mean refers to daily mean temperature [(max + min)/2], where max and min are the highest and lowest temperature, respectively, recorded at a station on a given day regardless of observation time. ^***b***^June–August. ^***c***^Based on daily mean temperature [(max + min)/2]; percentile is defined based on all calendar months.								

An inherent assumption with this approach is that heat impacts are relative depending on climate, an assumption that is widely supported in the literature (Braga et al. 2001, 2002; Davis et al. 2003a, 2003b; Donaldson et al. 2003; Kalkstein and Davis 1989; Medina-Ramón and Schwartz 2007; Pattenden et al. 2003). It is noteworthy that the diurnal changes in RR present in three northern cities (Boston, Philadelphia, and Seattle) are also evident in St. Louis, a location historically associated with high heat mortality (Medina-Ramón and Schwartz 2007), even though our results show a significant effect only at lag 0. Perhaps these northern cities lack the infrastructure (e.g., building materials, mechanical cooling systems) to fully mitigate heat effects in the afternoon and early evening. Although one might argue that the older housing stock built during times of historically cooler climates is less adaptable to heat impacts, the urban heat island effect, in which higher temperatures are observed in developed versus rural areas, has a much greater impact on minimum than maximum temperatures (Oke 1987).

The lack of strong or consistent relationships between temperature and all-cause mortality in Atlanta and Phoenix is supported by prior research showing a muted impact in warmer climates (Anderson and Bell 2009; Davis et al. 2003a, 2003b; Kalkstein and Davis 1989). Here, air conditioning and other cooling mechanisms (such as evaporative coolers, in the case of Phoenix) are prevalent, some infrastructure (e.g., buildings, parks, walkways) is designed to mitigate summer heat, and the populations have become acclimated (Chuang et al. 2013; Hartz et al. 2013). In Phoenix, it is noteworthy that the strongest mortality relationships at lag 0 and lag 1 are for the early morning and mid-morning hours and not the afternoon or evening. This finding is particularly relevant for efforts to diminish the climatic effects of future urbanization in the Phoenix metropolitan area, where mortality projections show wide disparities between maximum and minimum temperatures, the latter of which is primarily impacted by the urban heat island effect (Georgescu et al. 2011; Hondula et al. 2014). Because these cities experience potentially dangerous heat on a more routine basis than the other study cities, choice of a lower baseline temperature percentile (e.g., the 75th or 80th percentile) and/or choice of a different response variable (e.g., deaths specifically coded as heat related) may have revealed an association between heat and health undetected by the approach we used. In Phoenix, for example, the association between temperature and mortality has been shown to be stronger for models based on heat-related deaths versus those based on all-cause mortality (Petitti et al. 2015).

The results for Minneapolis-St. Paul are inconsistent with expectations. Although there are some statistically significant heat–mortality associations at lag 0 (e.g., RR at 0600 hours LST = 1.039; 95% CI: 1.009, 1.069) and late evening at lag 1 (e.g., RR at 2000 hours LST = 1.033; 95% CI: 1.005, 1.062), the overall mortality response was much less evident than in Boston, Philadephia, or Seattle. Other research has identified a demonstrable heat impact in Minneapolis using different periods of record and methods (Anderson and Bell 2009; Davis et al. 2003b; Hondula et al. 2015), with varying effect estimates for different demographic groups reported elsewhere (O’Neill et al. 2003, 2005). It may also be noteworthy that the somewhat shorter period of record in Minneapolis may have resulted in less robust RRs and larger confidence intervals compared with the other cities in this research.With respect to the daily summary temperature metrics, the differences in effect estimates tended to be minimal. Thus, based on this sample of cities, the impact of different methods of calculating the mean, maximum, and minimum temperature is small, and researchers are therefore encouraged to use the most convenient method. However, the selection of the daily metric to use—mean, maximum, or minimum—is more critical owing to differences in the estimated RR of mortality across the different cities we examined.

We observed that the times and days associated with the optimal cross-validation scores were not always the same as those that were associated with the largest model-estimated RR values. These two values are not directly comparable because they are calculated using different portions of the temperature distribution. RR is based on only the 99th and 85th percentile temperatures, whereas the cross-validation scores use the entire distribution. Models that do not provide a good fit across the entire temperature distribution but have a steep slope at high temperatures will appear to provide contrasting information between the RR and cross-validation scores. It is also possible that certain models are driven by a small number of highly influential data points that, when removed for subsetting the data for cross validation, lead to poorer model fit.

The temperature metrics we evaluated are often considered to be highly correlated with one another; if so, the differences in the shape of the temperature–mortality curves derived from different metrics (Figure 2) and the associated relative risk point estimates and cross-validation scores associated with different metrics (Figures 3–5) may seem to be surprisingly large. The extent to which these temperature metrics are, in fact, highly correlated with one another, depends on the portion of the temperature distribution under consideration. When examined from an annual perspective, most temperature observations and metrics are correlated because of seasonality; all metrics follow a similar progression throughout the course of the year. But differences between these metrics at the high end of the temperature distribution—those associated with summertime and extreme heat conditions—are most important in determining the RR estimates examined here. Temperature metrics are not as highly correlated at the extremes as they are for the entire summer or calendar year because the influence of seasonality becomes muted. For example, in comparing the overall correlations from all days during the summer months (~ 92 days) to the correlations from only those days with daily mean temperatures [(max + min)/2] above the 85th percentile of daily means (~ 55 days), the correlation between 1200 and 1800 hours LST temperatures (averaged across all cities) dropped from 0.829 to 0.615 (Table 3). Similarly, the correlation between daily maximum and minimum temperature declined from 0.702 to 0.417. The differences between these time series are carried forward into temperature–mortality models and can be manifested as large differences in the estimated temperature effect (as seen, for example, in Figure 2 and RR estimates in Figures 3 and 4).

Our results depend on accurate reporting of the date of death in the mortality records. We cannot control for possible inter-city biases associated with reporting practices, but we assume that any errors are randomly distributed. Because air temperature is highly autocorrelated, the hourly models are not independent. Although this would generally be a concern in the development of statistical models, it is not an issue in this research because our only goal was to compare temperature–mortality models at each hour. To allow for a quantitative comparison between cities and different temperature observation times and daily summary metrics, we selected consistent values for the percentiles of temperature used as the exposure contrast for the RRs (metric-specific 99th percentile vs. 85th percentile defined based on year-round data) and degrees of freedom in the smoothing splines (*k* = 7). The temperature percentiles we examined may not be the most appropriate representations of baseline and extreme heat conditions across all cities and observation times, and the absolute differences between the percentiles we examined certainly vary from one city to another and one time of day to another. Comparisons using other temperatures and splines influenced the point estimates and CIs of some the models but not the general shape of the curves (data not shown). Point estimates using the 90th percentile temperature metrics as the baseline were smaller and a higher number of CIs using the 90th percentile included the null, but the progression of estimates from one observation to another remained similar. Differences between the models with 5 or 7 degrees of freedom were generally minor and showed no systematic pattern toward larger or smaller point estimates or CIs. The siting of the meteorological stations used in this study may introduce some bias into the results, particularly because some of the stations are located in cities with a strong urban heat island effect (which impacts the diurnal temperature range) and/or one that has amplified over the study period (e.g., Chow et al. 2012; Hondula et al. 2012). We anticipate that any such bias would be small in light of other studies that have reported that temperature–mortality models using single meteorological stations perform just as well as those that incorporate multiple meteorological stations from different locations across urban areas (Guo et al. 2013; Schaeffer et al. 2016). Additional research is required to understand how well airport observations correlate to actual temperatures experienced by urban residents (e.g., Bernhard et al. 2015; Kuras et al. 2015). Despite the relatively long temporal records of mortality, sample size differences between cities may have influenced precision of effect estimates, although we did not observe any large biases related to sample size. Finally, because each day is examined independently, this study design does not allow us to specifically examine mortality displacement or the impacts of high heat over several consecutive days.

To our knowledge, this research is the first to examine the relationship between hour-by-hour temperature variations and temperature variable selection on heat-related mortality in the United States. The examination of hourly differences (Figures 3 and 4) provides a novel way of characterizing how the association between temperature and mortality evolves over fine time scales between the day of death and exposure 3 days prior. Cross-validation metrics indicate that models using temperatures from lag 0, and especially daytime hours on lag 0, were better predictors of excess mortality than other available options.

## Conclusions

After comparing 24 hourly temperatures and several different methods of calculating daily mean, maximum, and minimum temperature at lags of up to 3 days, we found differences in mortality effect estimates based solely on temperature variable selection in three cities commonly considered to be heat sensitive (Boston, Philadelphia, and Seattle). In these cities, afternoon, maximum, and daily mean temperatures were most strongly related to mortality, primarily at lag 0 (i.e., high heat occurring on the day of death). However, in cities where high summer temperatures are common (Phoenix, St. Louis, Atlanta), the relationships were weaker, and there was some evidence of a greater influence of morning temperatures at lag 0. In all cities, the relationships weakened significantly on or after lag 1, and some cities showed evidence of a mortality displacement effect by lag 3. In some cases, the statistical significance of effect estimates for mean, maximum, and minimum temperature depended on the manner in which the value was measured or calculated. In general, however, daily mean or maximum temperatures were more strongly associated with mortality than minimum temperatures, and differences between relative risks based on alternative versions of the same temperature metric (e.g., between maximum temperature based only on hourly measurements vs. the maximum temperature measured at any time during the day) were much smaller than differences between relative risks estimated for different temperature metrics (e.g., maximum vs. minimum daily temperature).

The lack of a single, consistent relationship across cities is consistent with prior research (Barnett et al. 2010; Hajat et al. 2010) and argues for the development of city-specific models. Based on these findings for a sample of U.S. cities from different climate zones, we recommend rigorous consideration and precise articulation of independent variable choice as well as examination of a number of alternative metrics (especially given the ready availability of hourly weather observations) when performing population-scale health risk assessment related to heat stress.
